# Neutrophil-to-albumin ratio predicts 30-day mortality in acute aortic dissection: a retrospective cohort study

**DOI:** 10.3389/fcvm.2026.1791503

**Published:** 2026-04-21

**Authors:** Yunxian Chen, Cheng Ling, Yang Song, Jiefan Xiao, Baofeng Chen, Liangqiu Tang, Shebing Zhang

**Affiliations:** Department of Cardiology, Yue Bei People’s Hospital, Shantou University Medical College, Shaoguan, China

**Keywords:** 30-day mortality, acute aortic dissection (ADD), inflammatory biomarkers, neutrophil-to-albumin ratio (NPAR), risk stratification

## Abstract

**Background:**

Acute aortic dissection (AAD) carries high early mortality, necessitating reliable prognostic tools. The neutrophil-to-albumin ratio (NPAR) synergistically encapsulates inflammatory activity and nutritional status, yet its prognostic utility in AAD remains underexplored.

**Methods:**

We retrospectively enrolled 415 patients with AAD diagnosed at Yuebei People's Hospital between January 2020 and December 2024. NPAR was calculated from admission laboratory values and analyzed as both a continuous and categorical variable (tertiles). Logistic regression models were used to assess the association between NPAR and 30-day mortality. Restricted cubic spline (RCS) analysis examined the dose–response relationship. Receiver operating characteristic analysis compared NPAR with NLR, SII, and PLR. Prespecified subgroup analyses examined effect consistency.

**Results:**

Higher NPAR values were independently associated with increased 30-day mortality (adjusted OR: 1.48; 95% CI: 1.05–2.09; *P* = 0.027). Patients in the highest NPAR tertile (≥22.37) had significantly greater mortality risk than those in the lowest tertile (<19.95) (adjusted OR: 20.28; 95% CI: 1.45–283.0; *P* = 0.025). RCS analysis revealed a linear positive association between NPAR and mortality. NPAR demonstrated superior discrimination for 30-day mortality (AUC: 0.741) compared with NLR, SII, and PLR. Subgroup analyses yielded consistent associations across prespecified strata with no significant interactions.

**Conclusions:**

Elevated admission NPAR is an independent predictor of short-term mortality in AAD. As an easily available and inexpensive biomarker, NPAR may improve early risk stratification and guide clinical decision-making in this high-risk population.

## GRAPHICAL ABSTRACT

**Figure:**
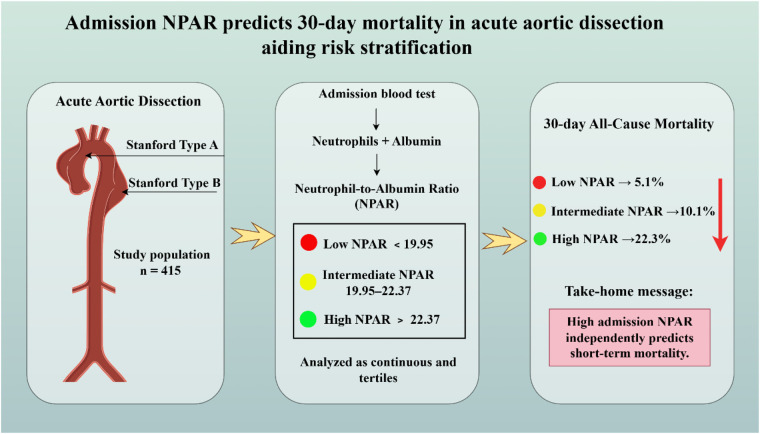

## 1 Introduction

Acute aortic dissection (AAD) is a life-threatening cardiovascular emergency caused by a tear in the aortic intima that creates a false lumen. According to the Stanford classification, type A dissection involves the ascending aorta, whereas type B affects the descending aorta (1). The overall mean annual incidence rate of aortic dissections was 4.2/100,000 patient-years, with type A aortic dissections (TAAD) at 2.2/100,000 and type B (TBAD) at 1.5/100,000 (2). Type A dissection carries a markedly higher mortality risk, increasing by 1%–2% per hour in the absence of timely surgical intervention. In contrast, type B dissections are generally managed conservatively, with surgery reserved for complicated presentations such as rupture, malperfusion, or refractory pain (3, 4). Despite advances in surgical and medical management, in-hospital mortality remains high—10%–20% for type A and 2.6%–16.1% for type B depending on complications (5, 6).

The pathogenesis of AAD involves both mechanical stress and inflammatory processes. Dissection triggers a systemic inflammatory response characterized by elevated biomarkers such as white blood cell count, D-dimer, and cytokine storms (IL-1β, IL-6, TNF-α) (7, 8). These markers have been linked to poorer outcomes, suggesting that inflammation contributes to disease progression. Hypoalbuminemia, a negative acute-phase reactant, has also been independently associated with increased mortality, underscoring the prognostic significance of both inflammation and nutritional status (9).

Recently, the neutrophil-to-albumin ratio (NPAR) has emerged as a novel biomarker that integrates inflammatory and nutritional information. Elevated NPAR has been associated with adverse outcomes in various cardiovascular disorders, including heart failure and acute coronary syndrome (10, 11). However, its prognostic role in AAD remains uncertain. Given the urgent need for early risk stratification, we hypothesised that admission NPAR could predict 30-day all-cause mortality in AAD, providing a simple and cost-effective tool to improve clinical decision-making and patient outcomes.

## 2 Methods

### 2.1 Study design and population

This single-centre retrospective cohort study was conducted at Yuebei People's Hospital, a tertiary cardiac centre in northern Guangdong Province, China. Consecutive adult patients admitted with acute aortic dissection between 1 January 2020 and 31 December 2024 were screened. The diagnosis of AAD was confirmed by imaging—contrast-enhanced computed tomography or magnetic resonance imaging—and standard diagnostic criteria (1). Patients with chronic dissection (symptom onset >14 days), traumatic dissection, haematologic abnormalities, active infection, known malignancy, or other severe systemic inflammatory conditions were excluded.

Dissections involving the ascending aorta (with or without descending extension) were classified as Stanford type A (type I), and those confined to the descending aorta as type B (type II). The study was approved by the institutional ethics committee (Ethical Lot Number: KY-2022-181). Informed consent was waived owing to the retrospective nature of the analysis. All procedures adhered to relevant ethical standards.

### 2.2 Data collection

Baseline demographic and clinical data were extracted from medical records, including age, sex, height, weight, blood pressure, presenting symptoms, dissection type, and comorbidities (hypertension, coronary artery disease, diabetes, chronic kidney disease, stroke, and peripheral vascular disease). Admission laboratory values were collected within 24 h, encompassing white blood cell, neutrophil, lymphocyte, and platelet counts; hemoglobin; creatinine; blood urea nitrogen; uric acid; corrected calcium; phosphorus; albumin; bilirubin; lipid profile; glucose; and potassium.

Derived indices included the neutrophil-to-lymphocyte ratio (NLR), platelet-to-lymphocyte ratio (PLR), and systemic inflammatory index (SII = platelet × neutrophil/lymphocyte). The NPAR was calculated as: [Neutrophils percentage (%) × 100/Albumin (g/dL)].

### 2.3 Study endpoint

The primary endpoint was all-cause mortality within 30 days of symptom onset. Follow-up was performed routinely for all discharged patients within 30 days.

### 2.4 Statistical analysis

Participants were stratified into tertiles according to their NPAR. Continuous variables were expressed as mean ± standard deviation and compared using one-way analysis of variance, whereas categorical variables were presented as counts (percentages) and analyzed with the *χ*^2^ or Fisher's exact test.

The association between NPAR and 30-day all-cause mortality was assessed using logistic regression, treating NPAR as both a continuous variable and by tertiles. The unadjusted model evaluated crude associations. Model I was adjusted for demographic and clinical covariates, including age, sex, smoking status, drinking habits, body mass index, systolic and diastolic blood pressure, heart rate, and major comorbidities (hypertension, diabetes mellitus, coronary artery disease, stroke, chronic kidney disease, and peripheral vascular disease). Model II further adjusted for potential confounders that altered the odds ratio by ≥10%, including white blood cell count, hemoglobin, creatine kinase, CK-MB, troponin T, creatinine, Stanford type, surgical treatment, neutrophil-to-lymphocyte ratio (NLR), platelet-to-lymphocyte ratio (PLR), and systemic inflammatory index (SII).

Restricted cubic spline regression assessed potential non-linearity in the NPAR–mortality association, using the median NPAR as the reference. Receiver operating characteristic (ROC) curve analyses quantified the discriminatory ability of NPAR, NLR, PLR, and SII, with optimal cut-off values derived from the Youden index. Subgroup analyses explored effect modification across key clinical strata, and interaction terms were tested for heterogeneity. A two-tailed *P* < 0.05 was considered statistically significant. All analyses were conducted using SPSS version 26.0 (IBM Corp.) and R version 4.1.

## 3 Results

### 3.1 Population and baseline characteristics

**Figure 1:**
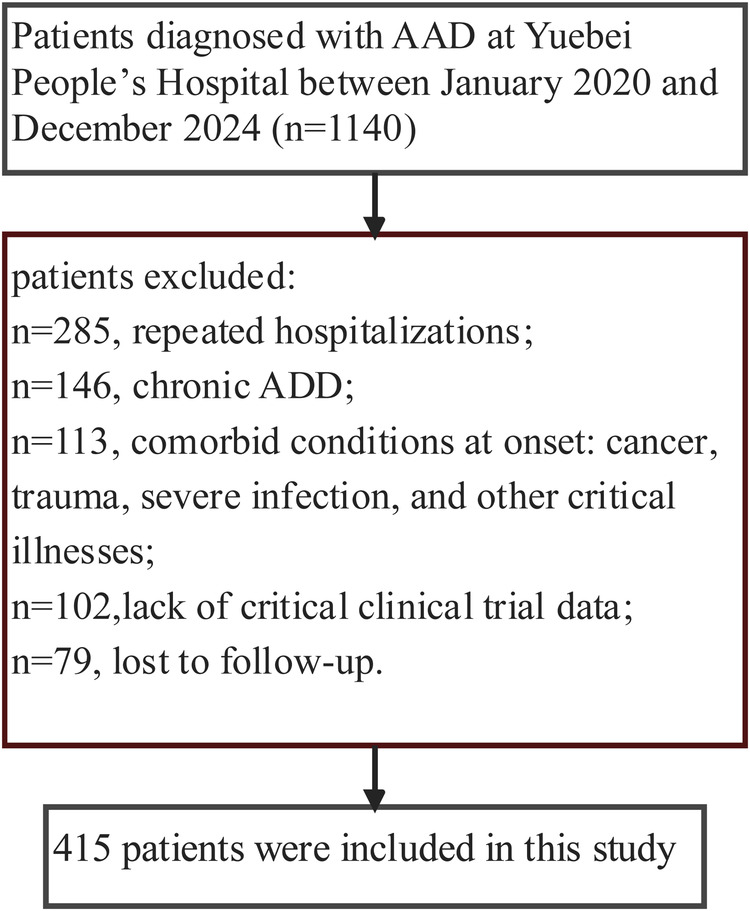
Flowchart of patient selection.

After excluding ineligible cases, 415 patients were included (Figure 1). Baseline characteristics are summarised in Table 1. Patients in higher NPAR tertiles were older (*P* = 0.013) and had lower diastolic blood pressure (*P* = 0.027). Sex distribution, body mass index, and prevalence of hypertension, diabetes, and coronary artery disease were comparable among tertiles.

**Table 1:** Baseline characteristics of the study participants.

Variables	Total (*n* = 415)	Tertile 1 (*n* = 138) ＜19.95	Tertile 2 (*n* = 138) 19.95-22.37	Tertile 3 (*n* = 139) ＞22.37	*p*
Age (years)	59.7 ± 12.1	58.1 ± 12.8	58.9 ± 11.8	62.1 ± 11.5	0.013
Male, *n* (%)	332 (80.0)	114 (82.6)	111 (80.4)	107 (77)	0.497
BMI (Kg/m^3^)	24.9 ± 3.9	25.0 ± 4.1	25.3 ± 3.6	24.3 ± 4.0	0.287
Smoking, *n* (%)	107 (25.8)	42 (30.4)	30 (21.7)	35 (25.2)	0.251
Drinking, *n* (%)	55 (13.3)	23 (16.7)	15 (10.9)	17 (12.2)	0.332
Vital signs upon admission					
SBP (mmHg)	160.8 ± 31.7	164.2 ± 30.7	163.1 ± 30.9	155.1 ± 32.8	0.07
DBP (mmHg)	90.8 ± 19.9	94.6 ± 20.6	90.6 ± 18.8	87.4 ± 19.6	0.027
HR (bpm)	80.5 ± 14.8	80.1 ± 14.3	80.7 ± 14.0	80.5 ± 16.1	0.955
Comorbidities, *n* (%)					
Hypertension	384 (92.5)	124 (89.9)	130 (94.2)	130 (93.5)	0.335
Diabetes	18 (4.3)	5 (3.6)	3 (2.2)	10 (7.2)	0.107
Coronary artery disease	42 (10.1)	16 (11.6)	13 (9.4)	13 (9.4)	0.781
Stroke	66 (15.9)	21 (15.2)	18 (13)	27 (19.4)	0.336
Chronic kidney disease	64 (15.4)	22 (15.9)	14 (10.1)	28 (20.1)	0.069
Peripheral vascular disease	143 (34.5)	47 (34.1)	44 (31.9)	52 (37.4)	0.622
Laboratory parameters					
WBC (×10^9^/L)	12.0 ± 4.4	10.6 ± 4.1	12.2 ± 3.8	13.2 ± 4.8	<0.001
Neutrophil Count (×10^9^/L)	10.1 ± 4.3	8.2 ± 3.9	10.4 ± 3.7	11.6 ± 4.6	<0.001
Lymphocyte count (×10^9^/L)	1.1 ± 0.5	1.4 ± 0.5	1.1 ± 0.5	0.9 ± 0.4	<0.001
Neutrophil percentage (%)	82.3 ± 8.9	75.6 ± 9.5	83.8 ± 7.1	87.5 ± 5.1	<0.001
Lymphocyte percentage (%)	10.4 ± 6.7	15.1 ± 8.0	9.4 ± 5.0	6.6 ± 3.1	<0.001
Hemoglobin (g/L)	126.3 ± 19.3	131.8 ± 18.5	128.0 ± 16.9	119.1 ± 20.2	<0.001
Platelet (×10^9^/L)	199.2 ± 71.7	211.2 ± 67.9	193.0 ± 67.4	193.3 ± 78.1	0.054
Potassium (mmol/L)	4.1 ± 0.6	4.1 ± 0.4	4.1 ± 0.5	4.3 ± 0.7	0.004
Sodium (mmol/L)	140.2 ± 3.4	140.5 ± 3.6	140.1 ± 3.0	140.1 ± 3.6	0.561
Uric acid (μmol/L)	393.3 ± 129.7	392.0 ± 135.0	389.8 ± 125.7	398.3 ± 128.9	0.859
Blood urea nitrogen (mmol/L)	7.1 ± 4.4	6.4 ± 4.7	6.6 ± 2.9	8.2 ± 5.0	0.001
Glucose (mmol/L)	7.6 ± 2.8	6.9 ± 2.5	7.3 ± 2.3	8.4 ± 3.2	<0.001
Creatinine (μmol/L)	132.6 ± 160.1	124.0 ± 174.2	118.5 ± 138.5	155.5 ± 164.3	0.119
Tropotin T (pg/mL)	93.6 ± 341.6	51.4 ± 157.2	102.2 ± 436.5	125.9 ± 364.3	0.216
CK-MB (U/L)	20.0 ± 23.3	17.0 ± 13.6	22.1 ± 31.0	20.7 ± 21.5	0.209
CK (U/L)	298.3 ± 1237.3	203.9 ± 543.8	460.5 ± 1980.0	224.6 ± 522.3	0.185
AST (U/L)	45.1 ± 202.1	31.5 ± 58.6	27.1 ± 29.5	75.9 ± 340.2	0.086
ALT (U/L)	37.1 ± 155.1	30.9 ± 51.7	22.4 ± 14.4	57.6 ± 260.8	0.145
Albumin (g/L)	39.0 ± 4.5	41.7 ± 4.0	39.6 ± 3.4	35.7 ± 3.7	<0.001
Cholesterol (mmol/L)	4.7 ± 0.9	4.8 ± 0.9	4.6 ± 0.8	4.7 ± 1.0	0.142
LDL-C (mmol/L)	2.6 ± 0.8	2.7 ± 0.7	2.6 ± 0.7	2.5 ± 0.9	0.102
Stanford classification					0.004
Type A	136 (32.9)	32 (23.2)	46 (33.6)	58 (42)	
Type B	277 (67.1)	106 (76.8)	91 (66.4)	80 (58)	
Compound biomarkers					
NPAR	21.4 ± 3.2	18.2 ± 1.6	21.2 ± 0.7	24.7 ± 2.3	<0.001
NLR	11.7 ± 9.6	7.4 ± 5.7	12.4 ± 10.4	15.6 ± 10.2	<0.001
PLR	216.0 ± 131.2	181.3 ± 105.3	214.0 ± 131.6	254.9 ± 145.1	<0.001
SII	2200.2 ± 1672.8	1541.9 ± 1277.1	2202.6 ± 1546.6	2893.0 ± 1888.0	<0.001
Surgical treatment, *n* (%)	147 (35.4)	57 (41.3)	51 (37)	39 (28.1)	0.063
Length of hospital stay (days)	11.4 ± 9.3	11.2 ± 6.5	11.5 ± 9.9	11.6 ± 11.0	0.916
30-day mortality, *n* (%)	52 (12.5)	7 (5.1)	14 (10.1)	31 (22.3)	<0.001

Data were presented as *n* (%) and mean (SD). BMI, body mass idex; SBP, systolic blood pressure; DBP, diastolic blood pressure; HR, heart rate; WBC, white blood cell; CK-MB, heart-type isoenzyme of creatine kinase; CK, Creatine kinase; LDL-C, low-density lipoprotein cholesterol; NPAR, neutrophil percentage-to-albumin ratio; NLR, neutrophil-to-lymphocyte ratio; PLR, platelet-to-lymphocyte ratio; SII, systemic inflammatory index [(platelet count × neutrophil count)/lymphocyte count].

Laboratory parameters showed progressively higher white blood cell and neutrophil counts, glucose, and blood urea nitrogen, while albumin and hemoglobin decreased significantly with increasing NPAR (all *P* < 0.001). Stanford type A dissections were more common in the highest tertile (*P* = 0.004). Surgical intervention rates declined with increasing NPAR but did not reach statistical significance (*P* = 0.063). Importantly, 30-day mortality increased markedly across tertiles (5.1%, 10.1%, and 22.3%; *P* < 0.001), indicating a strong relationship between elevated NPAR and short-term adverse outcomes.

### 3.2 Association between NPAR and 30-day mortality

As shown in Table 2, higher NPAR levels were independently associated with increased 30-day all-cause mortality. When modelled as a continuous variable, each unit increase in NPAR was associated with higher mortality risk across all models (adjusted OR: 1.48; 95% CI: 1.05–2.09; *P* = 0.027 in Model II).

**Table 2:** Association between NPAR and 30-day all-cause mortality.

Variables	n.total	n.event_%	Non-adjusted Model		Model I		Model II	
			OR (95% CI)	*P*	OR (95% CI)	*P*	OR (95% CI)	*P*
NPAR	415	52 (12.5)	1.22 (1.12–1.34)	<0.001	1.24 (1.04–1.47)	0.017	1.48 (1.05–2.09)	0.027
NPAR tertiles								
＜19.95	138	7 (5.1)	1(Ref)		1(Ref)		1(Ref)	
19.95–22.37	138	14 (10.1)	2.11 (0.83–5.41)	0.119	2.9 (0.58–14.41)	0.193	4.89 (0.5–47.43)	0.171
＞22.37	139	31 (22.3)	5.37 (2.28–12.68)	<0.001	7.11 (1.63–31.03)	0.009	20.28 (1.45–283)	0.025

Data presented are ORs and 95% CIs. Adjust model I adjusts for age, gender, smoking, drinking, hypertension, diabetes, coronary artery disease, stroke, chronic kidney disease, peripheral vascular disease, SBP, DBP, HR, BMI; Adjusted Model II: In addition to the variables in Model I, adjust those variables whose inclusion in this model causes the odds ratio to change by at least 10%, including WBC, hemoglobin, CK, CK-MB, tropotin T, creatinine, stanford classification, surgical treatment, NLR, PLR, SII.

When analysed by tertiles, patients in the highest NPAR tertile (≥22.37) had significantly higher mortality than those in the lowest (<19.95) and intermediate (19.95–22.37) tertiles, with adjusted ORs of 20.28 (95% CI: 1.45–283.0; *P* = 0.025) and 15.67 (95% CI: 1.21–203.0; *P* = 0.034), respectively. No significant difference was observed between the lowest and intermediate tertiles (*P* > 0.05).

**Figure 2:**
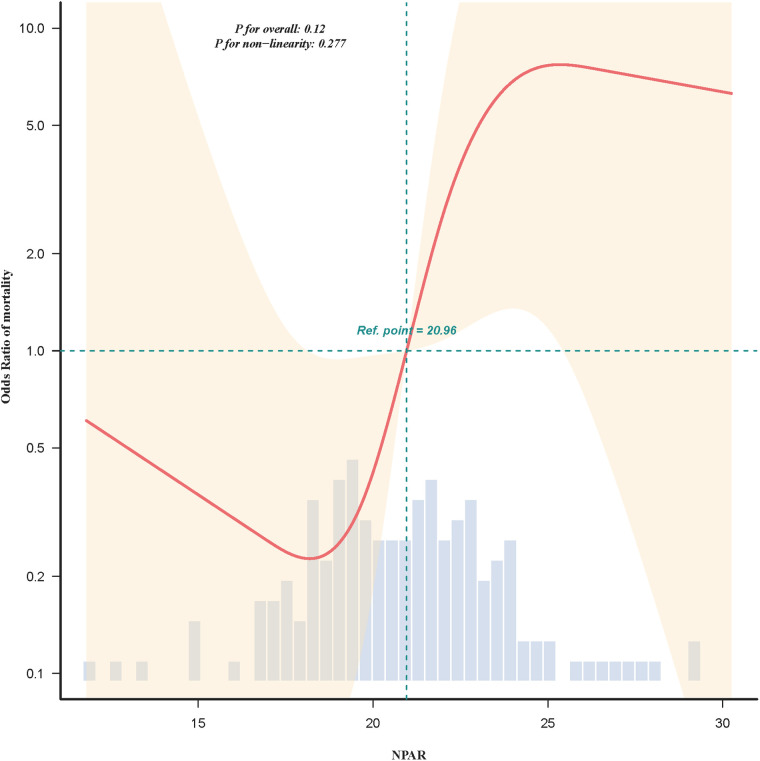
Restricted cubic spline (RCS) regression illustrating the adjusted relationship between NPAR and 30-day all-cause mortality. The solid line indicates the estimated odds ratio (OR) for mortality, and the shaded area represents the 95% confidence interval (CI). The reference point was set at the median NPAR value (20.96).The model was adjusted for all covariates included in Model II of Table 2. The spline curve demonstrates a positive, approximately linear association between NPAR and 30-day mortality (*P* for overall = 0.12; *P* for non-linearity = 0.277).

Restricted cubic spline analysis (Figure 2) demonstrated a positive, approximately linear association between NPAR and mortality risk (*P* for non-linearity = 0.28). The predicted probability of death increased steadily with rising NPAR, particularly above 22, and remained consistent after full multivariable adjustment.

### 3.3 ROC and subgroup analyses

**Figure 3:**
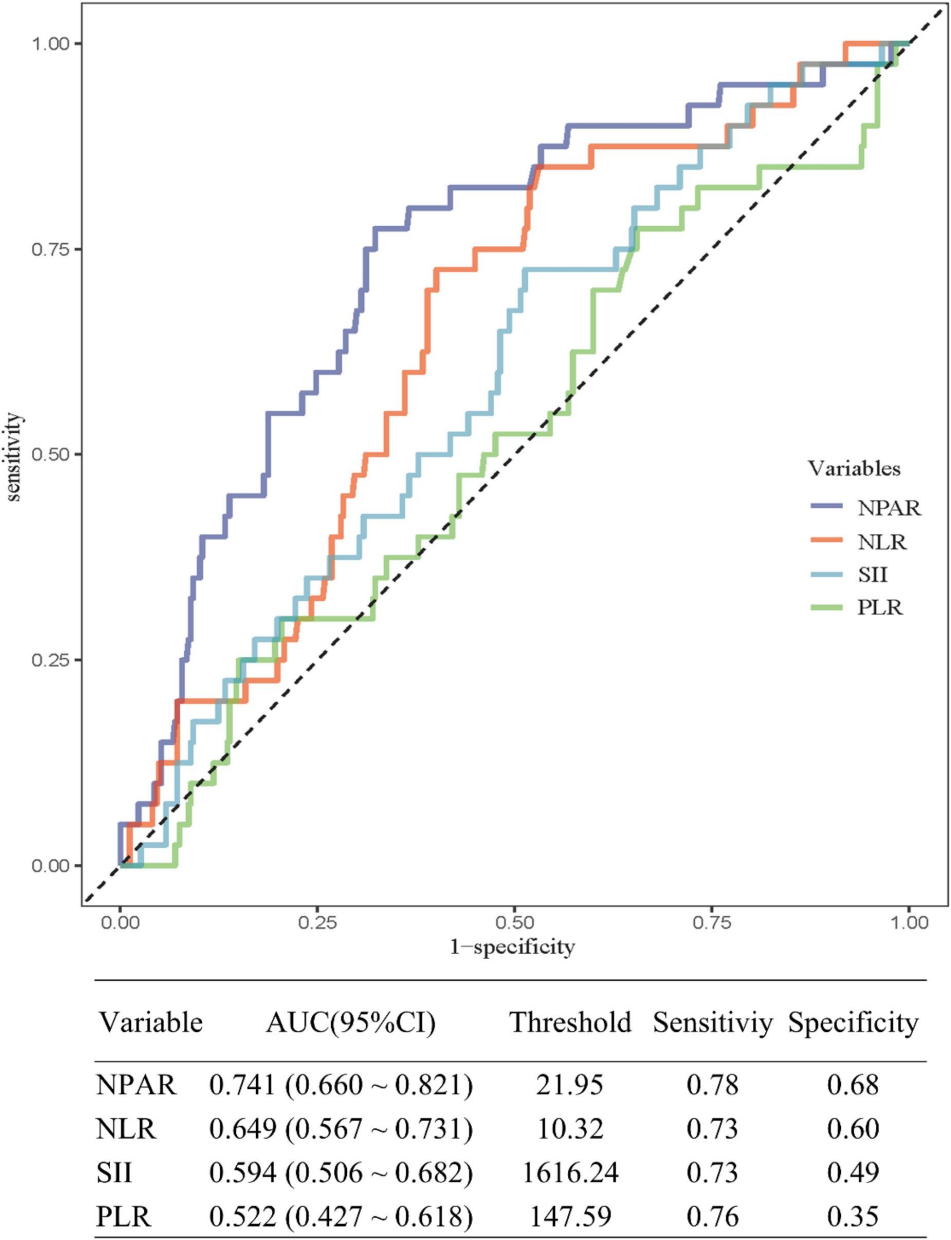
ROC curves for NPAR and other inflammatory markers in predicting 30-day all-cause mortality in ADD patients, along with AUC, threshold values, sensitivity, and specificity across groups.

ROC analysis (Figure 3) showed that NPAR had the highest discriminatory ability for predicting 30-day mortality (AUC: 0.741; 95% CI: 0.660–0.821), with an optimal threshold of 21.95 (sensitivity 78%, specificity 68%). NLR (AUC: 0.649), SII (0.594), and PLR (0.522) demonstrated weaker discrimination.

**Figure 4:**
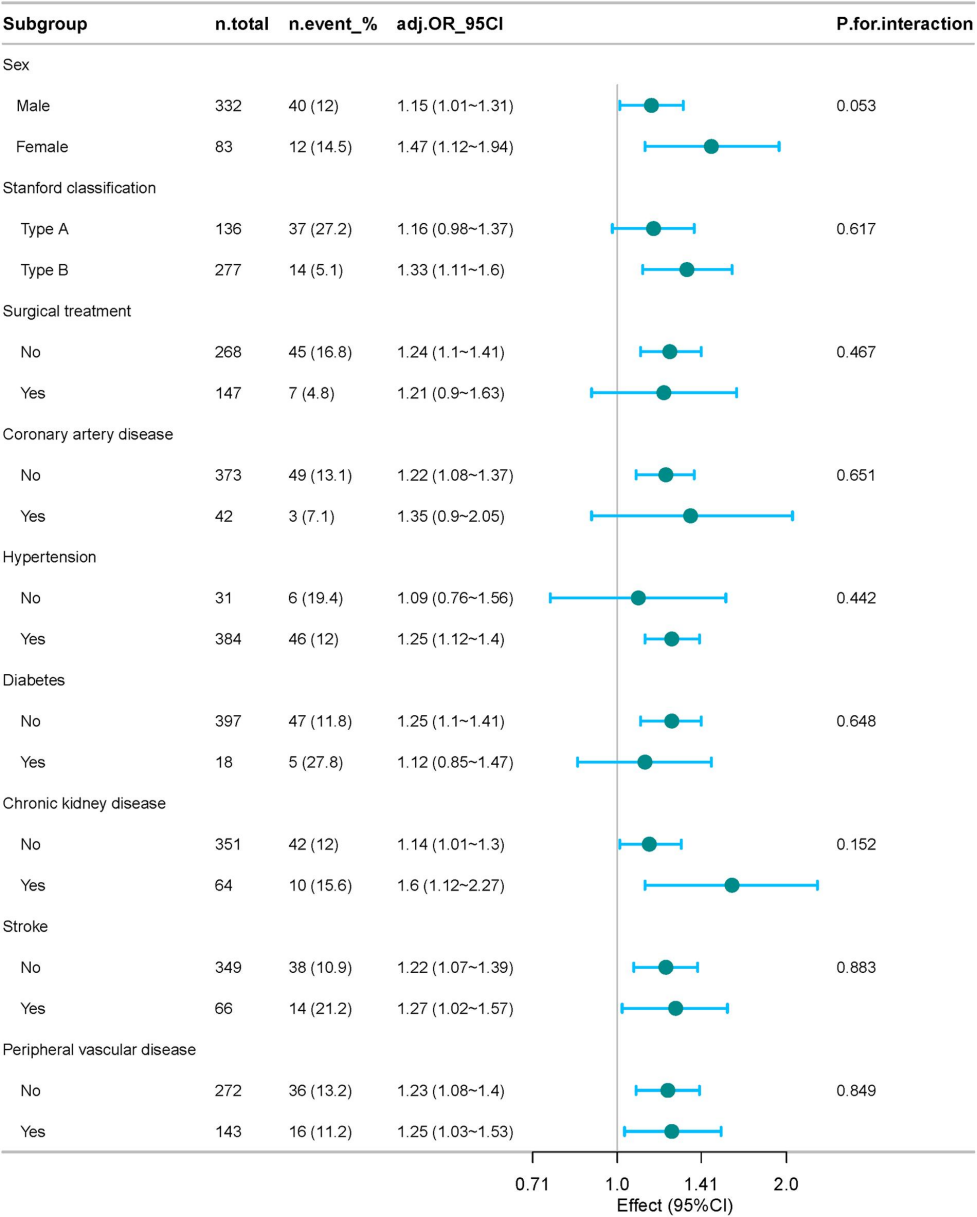
Forest plot showing the results of subgroup analyses assessing the relationship between NPAR (per unit increase) and 30-day all-cause mortality. Multivariable logistic regression models were adjusted for the same covariates as Model II in Table 2. Overall, the robustness of the association across diverse clinical subgroups underscores the reliability of NPAR as an independent predictor of short-term mortality in acute aortic dissection.

Subgroup analyses (Figure 4) confirmed the robustness of the association between elevated NPAR and mortality across most strata. Adjusted ORs (per unit increase) ranged from 1.09 to 1.60, without significant effect modification (all *P* for interaction >0.05). A borderline sex interaction (*P* = 0.053) suggested a potentially stronger association in females. Overall, the findings indicate that high NPAR consistently predicts increased short-term mortality in ADD.

## 4 Discussion

In this retrospective cohort study, we identified the NPAR—a composite marker integrating systemic inflammation and nutritional status—as a strong and independent predictor of 30-day all-cause mortality in patients with AAD. The relationship between NPAR and mortality was linear and remained consistent across all subgroups, even after comprehensive adjustment for clinical and biochemical confounders. Among the inflammatory indices evaluated, NPAR demonstrated the highest discriminatory performance for short-term mortality.

By combining neutrophil count and serum albumin, NPAR captures complementary physiological information that isolated biomarkers cannot provide. Previous studies have shown that both heightened inflammatory activity and hypoalbuminaemia are individually associated with adverse outcomes in AAD, but neither offers robust prognostic precision. NPAR bridges these dimensions—acute inflammatory activation and diminished metabolic reserve—into a single, physiologically meaningful parameter. Our findings align with growing evidence that composite inflammatory-nutritional indices outperform single markers in predicting outcomes in cardiovascular emergencies.

### 4.1 Pathophysiological mechanisms

The pathogenesis of AAD involves acute mechanical disruption of the aortic intima, initiating a profound local and systemic inflammatory response. Neutrophils rapidly infiltrate the aortic wall, releasing proteolytic enzymes, reactive oxygen species, and pro-inflammatory cytokines that exacerbate tissue injury and endothelial dysfunction (12, 13). Patients with AAD typically present with elevated white blood cell and neutrophil counts, as well as higher neutrophil-to-lymphocyte ratios, highlighting the central role of neutrophil-driven inflammation in early mortality (14, 15).

Conversely, serum albumin is a well-recognized marker of both nutritional status and systemic inflammation (16). Hypoalbuminaemia results from increased vascular permeability, cytokine-mediated suppression of hepatic synthesis, and fluid redistribution during acute inflammatory states (17). Low albumin concentrations have been associated with adverse outcomes in multiple cardiovascular disorders, including heart failure and acute coronary syndromes, where they predict in-hospital mortality and incident heart failure (18, 19). Moreover, in patients with type B AAD, hypoalbuminaemia has been linked to increased long-term mortality (20). The combined presence of neutrophilia and hypoalbuminaemia therefore encapsulates both inflammatory burden and metabolic fragility, providing a biologically plausible basis for the prognostic power of NPAR.

Although the molecular mechanisms connecting neutrophil activation and albumin depletion to poor outcomes remain incompletely elucidated, experimental evidence implicates neutrophil extracellular traps, matrix metalloproteinase activity, and endothelial injury as potential mediators (21). Future translational studies are warranted to delineate these mechanisms and to explore whether modulating inflammation or improving nutritional status can alter clinical outcomes.

### 4.2 Comparison with previous biomarker studies

Our findings complement and extend prior work on inflammatory biomarkers in AAD. Several studies have evaluated the prognostic value of the neutrophil-to-lymphocyte ratio (NLR), platelet-to-lymphocyte ratio (PLR), and systemic immune-inflammation index (SII) across cardiovascular diseases, including atherosclerosis (22, 23), coronary heart disease (24, 25), and heart failure (26, 27). In AAD, elevated NLR and PLR have been associated with higher in-hospital mortality (28), with reported AUC values ranging from 0.60 to 0.75, reflecting modest but inconsistent predictive ability (29). In surgical cohorts of acute type A aortic dissection (ATAAD), increased preoperative SII has been independently associated with 30-day mortality (OR: 3.532; 95% CI: 1.719–7.255; *P* = 0.001) (30).

Albumin-based indices such as the prognostic nutritional index (PNI) have also demonstrated prognostic value in AAD (31), reinforcing the importance of combining inflammatory and nutritional markers. A prior study focusing on surgical ATAAD reported that patients in the upper NPAR tertile had significantly higher in-hospital mortality (AUC: 0.708) (29). Our study expands these findings by including both Stanford type A and B dissections, incorporating surgically and medically managed patients, and extending observation to 30 days. The higher AUC observed here (0.741) and the steeper mortality gradient among tertiles likely reflect broader case inclusion and outcome definitions. Notably, few investigations have directly compared NPAR, NLR, PLR, and SII within the same cohort. Our results suggest that NPAR provides superior predictive accuracy for short-term mortality, consistent across both type A and type B dissections and irrespective of treatment modality.

### 4.3 Clinical utility

From a clinical perspective, the primary advantage of NPAR lies in its simplicity, accessibility, and cost-effectiveness. It can be rapidly calculated from standard admission laboratory tests without additional assays, making it particularly valuable in emergency and resource-limited settings. Moreover, NPAR may help guide future trials exploring anti-inflammatory or nutritional optimization strategies in high-risk AAD populations. While our data support its prognostic value, the potential of NPAR as a modifiable therapeutic target warrants prospective investigation.

### 4.4 Study limitations

This study has several limitations. First, it was conducted at a single centre with a retrospective design, which may limit external generalisability. Validation in multicentre, prospective cohorts is needed. Second, NPAR was calculated from a single admission sample; temporal trends or serial measurements could provide additional insights into disease dynamics. Third, despite extensive covariate adjustment, residual confounding from unmeasured variables such as nutritional status or chronic inflammation cannot be excluded. Fourth, we employed tertile-based grouping to illustrate risk gradients, but the optimal clinical cut-off for NPAR remains undetermined. Future studies should define standardized thresholds using calibration and decision-curve analyses. Finally, our study focused on 30-day mortality; longer-term outcomes and postoperative complications were not assessed.

## 5 Conclusions

In summary, the NPAR is a simple, readily obtainable biomarker that independently predicts 30-day mortality in patients with ADD. By integrating markers of inflammatory activation and nutritional reserve, NPAR offers a biologically sound and practical tool for early risk stratification. Incorporating NPAR into clinical decision frameworks may enhance prognostic accuracy, optimise triage, and ultimately improve outcomes in this life-threatening condition.

## Data Availability

The original contributions presented in the study are included in the article/Supplementary Material, further inquiries can be directed to the corresponding author.
